# Discovering the Context of People With Disabilities: Semantic Categorization Test and Environmental Factors Mapping of Word Embeddings from Reddit

**DOI:** 10.2196/17903

**Published:** 2020-11-20

**Authors:** Alejandro Garcia-Rudolph, Joan Saurí, Blanca Cegarra, Montserrat Bernabeu Guitart

**Affiliations:** 1 Institut Guttmann Hospital de Neurorehabilitacio Badalona Spain; 2 Universitat Autònoma de Barcelona Bellaterra (Cerdanyola del Vallès) Spain; 3 Fundació Institut d’Investigació en Ciències de la Salut Germans Trias i Pujol Badalona Spain; 4 Universitat de Barcelona Barcelona Spain

**Keywords:** disability, Reddit, social media, word2vec, semantic categorization, silhouette, activities of daily life, aspects of daily life, context, embeddings

## Abstract

**Background:**

The World Health Organization’s International Classification of Functioning Disability and Health (ICF) conceptualizes disability not solely as a problem that resides in the individual, but as a health experience that occurs in a context. Word embeddings build on the idea that words that occur in similar contexts tend to have similar meanings. In spite of both sharing “context” as a key component, word embeddings have been scarcely applied in disability. In this work, we propose social media (particularly, Reddit) to link them.

**Objective:**

The objective of our study is to train a model for generating word associations using a small dataset (a subreddit on disability) able to retrieve meaningful content. This content will be formally validated and applied to the discovery of related terms in the corpus of the disability subreddit that represent the physical, social, and attitudinal environment (as defined by a formal framework like the ICF) of people with disabilities.

**Methods:**

Reddit data were collected from pushshift.io with the pushshiftr R package as a wrapper. A word2vec model was trained with the wordVectors R package using the disability subreddit comments, and a preliminary validation was performed using a subset of Mikolov analogies. We used Van Overschelde’s updated and expanded version of the Battig and Montague norms to perform a semantic categories test. Silhouette coefficients were calculated using cosine distance from the wordVectors R package. For each of the 5 ICF environmental factors (EF), we selected representative subcategories addressing different aspects of daily living (ADLs); then, for each subcategory, we identified specific terms extracted from their formal ICF definition and ran the word2vec model to generate their nearest semantic terms, validating the obtained nearest semantic terms using public evidence. Finally, we applied the model to a specific subcategory of an EF involved in a relevant use case in the field of rehabilitation.

**Results:**

We analyzed 96,314 comments posted between February 2009 and December 2019, by 10,411 Redditors. We trained word2vec and identified more than 30 analogies (eg, breakfast – 8 am + 8 pm = dinner). The semantic categorization test showed promising results over 60 categories; for example, s(A relative)=0.562, s(A sport)=0.475 provided remarkable explanations for low s values. We mapped the representative subcategories of all EF chapters and obtained the closest terms for each, which we confirmed with publications. This allowed immediate access (≤ 2 seconds) to the terms related to ADLs, ranging from apps “to know accessibility before you go” to adapted sports (boccia). For example, for the support and relationships EF subcategory, the closest term discovered by our model was “resilience,” recently regarded as a key feature of rehabilitation, not yet having one unified definition. Our model discovered 10 closest terms, which we validated with publications, contributing to the “resilience” definition.

**Conclusions:**

This study opens up interesting opportunities for the exploration and discovery of the use of a word2vec model that has been trained with a small disability dataset, leading to immediate, accurate, and often unknown (for authors, in many cases) terms related to ADLs within the ICF framework.

## Introduction

### General Background

Natural Language Processing (NLP) is increasingly being integrated into several application domains. Google AI recently introduced BERT (Bidirectional Encoder Representations from Transformers) [1] to match search queries with more relevant results for optimizing Google searches. Facebook AI also achieved impressive breakthroughs, such as by tackling harmful or improper content by means of Whole Post Integrity Embeddings (WPIE) [2]. Other examples can be found in mobile apps, such as virtual assistants like Amazon’s Alexa or Apple’s Siri [3]. Application domains range from cultural heritage [4] to the identification of concepts and relationships in a body of research papers [5] or clinical decision support systems [6].

Words that occur in similar contexts tend to have similar meanings. This was likely first formulated in 1954 by Harris [7]. But the most famous statement of this principle came a few years later from linguist JR Firth: “You shall know a word by the company it keeps!” [8].

One of the strongest trends in NLP at the moment is the use of word embeddings, which are vectors whose relative similarities correlate with semantic similarity, building on the ideas of Harris and Firth.

The approval of the International Classification of Functioning, Disability, and Health (ICF) [9] by the World Health Assembly in May 2001 has marked a paradigm shift in the way health and disability are understood and measured [10]. The ICF conceptualizes disability not solely as a problem that resides in the individual but as a health experience that occurs in a *context* [11].

Disability and functioning are, according to the ICF model, outcomes of interactions between health conditions (diseases, disorders, and injuries) and contextual factors [9].

In spite of both sharing context as a key component, word embeddings have been scarcely applied in the field of disability, to the best of our knowledge.

In this paper, we hypothesize that social media can, indeed, link them. Word embeddings are usually learned from a general-purpose corpus; when it doesn't match the domain's vocabulary (including the same words or using words in the same senses), it is a problem that cannot simply be fixed with a lot of data. More data could just pull word contexts and representations towards generic, rather than domain-specific, values.

Our hypotheses in this paper are the following: (1) Such domain-specific values can be extracted from public domain-specific social media (2) in a sufficient number for the embedding to be relevant to the ICF model and (3) verifiable by sound theoretical semantic tests (4) consistent with state-of-the-art publications and (5) providing actionable knowledge to onfield specialists.

### Social Media

Social media statistics from 2019 show that there are 3.2 billion social media users worldwide, and this number is growing [12].

Recent analyses remark that 42% of internet users take advantage of social media for health information, 32% of social media users in the United States share their health care experiences and family’s struggle stories, and 29% search for health information via social media platforms to observe others’ experiences with their diseases. Furthermore, 51% of those who live with a chronic disease have used the internet for information about health topics, such as details of a specific disease, medical procedures, drugs, medical devices, or health insurances [13].

#### Reddit

Social media users on platforms such as Reddit [14] tend to sharply contrast with similar groups that participate offline; for instance, people on Reddit are likely to discuss problems that they do not feel comfortable discussing face-to-face [15].

Another reason Reddit was chosen as a data source for this study is that the language of text posts is more structured than on other social media platforms such as Twitter [13].

As of 2019, Reddit’s official statistics included 430 million monthly active users, 199 million posts, 1.7 billion comments, and around 14 billion views in a single month [16].

Reddit’s core functionality is the sharing of text-based posts with others who may or may not be members of the site. The subforum function allows the creation of designated spaces for users to congregate and interact with each other over a shared interest. These subforums are called subreddits.

### This Study

In the following subsections, we describe the specific characteristics and objectives of this study.

#### The Disability Subreddit

**Figure 1:**
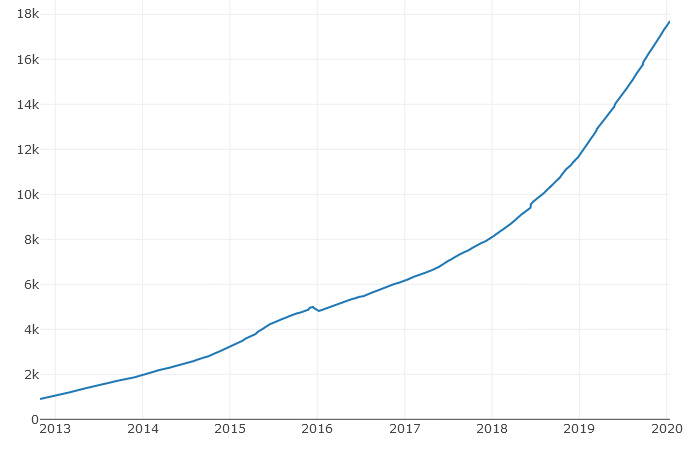
The number of subscribers in the disability subreddit by year.

The data used for this study were extracted from the disability subreddit (containing news, resources, and perspectives pertaining to individuals with disabilities). It numbers 17,545 subscribers and 17 comments per day [17]. The evolution of the number of subscribers since 2013 is shown in Figure 1. The disability subreddit was created on March 12, 2008.

The total number of posts and comments since 2008 are not shown in the Reddit official statistics; the 17 comments per day are, in fact, “the comments received on all its posts in a recent 24 hour measurement period. This number isn’t averaged over time” [17]. A plot of the comments per day during the November 2018-December 2019 period can also be obtained from the official statistics [17] (Multimedia Appendix 1). Therefore, taking as a starting point the total number of subscribers (Figure 1) and the total number of comments during the last year, a rough estimation leads to about 100,000 comments during the 2009-2019 time period.

#### Domain-Specific Values: Environmental Factors

According to the ICF, contextual factors represent the complete background of an individual’s life and living [18]. They include 2 components: environmental factors (EF) and personal factors, which may have an impact on an individual with a health condition and that individual’s health and health-related states.

In this study, we focus on the EF section of the ICF; by definition, EF make up the physical, social, and attitudinal environment in which people live and conduct their lives [9]. EF are organized into 5 chapters, each dealing with different and mutually exclusive aspects of the environment: (e1) products and technology, (e2) natural environment and human-made changes to the environment, (e3) support and relationships, (e4) attitudes, and (e5) services, systems, and policies.

#### Word2vec

Word2vec is the method used in this study for generating word embeddings. It creates an embedding (ie, numerical representations of words that help capture meaning, semantic relationships, and context) for text by using each word in a corpus to predict the words that usually surround it [19]. It consists of 2 neural network models: Continuous Bag of Words (CBOW) and Skip-gram. In both models, a window of predefined length is moved along the corpus, and in each step, the network is trained with the words inside the window to predict the word in the center of the window based on the surrounding words (CBOW) or to predict the contexts based on the central word (Skip-gram).

Therefore, word2vec creates word embeddings in which the semantic relationships between words are preserved. In this paper, we use the Skip-gram model, which shows better performance in semantic tasks [20].

#### Small Dataset: Twofold Validation of Word2vec Embedding

Word2vec methods have a distinct advantage in handling large datasets and have been trained with billions of tokens, as shown in the Google archive of the original Mikolov paper [21] (eg, the latest Wikipedia dumps 3 billion words, or the “one-billion-word language-modeling benchmark”).

As it is a prediction-based model, it might be reasonable to expect that word2vec will produce very low-quality embeddings when trained with a small corpus.

Nevertheless, based on the high specificity of the disability subreddit, we hypothesize that the produced embeddings will be of high semantic quality. In order to verify this, we will follow a twofold validation.

First, we will apply the semantic categorization test in order to measure the word2vec model’s capabilities of representing semantic categories (such as vegetables, countries, fruits, and clothes). The original test (the Battig and Montague norm [22]) is composed of 53 categories with 10 words each. In order to measure how well the word *i* is grouped in relation to the other words in its semantic category, we will use silhouette coefficients [23].

Second, for each EF chapter (e1-e5), we will map meaningful word embeddings to representative categories of each chapter and refer to relevant publications to confirm their practical value.

#### Study Objectives

Specific aspects of disability (eg, depression) have been studied on social platforms such as Twitter [24] or support groups for Autism Spectrum Disorder on Facebook [25]. However, to our knowledge, no published study has examined social media content related to the environmental factors that make up the physical, social, and attitudinal environment in which people with disabilities live and conduct their lives.

Therefore, in this study, we aim to do the following: (1) extract all comments and submissions from the disability subreddit during the period under study (2009-2019); (2) train a word2vec model using disability subreddit comments as a training set, performing a preliminary validation using a subset of the original Mikolov paper analogies; (3) perform a semantic categorization test using an updated and expanded version of the Battig and Montague norm, with 65 categories; for each category, compute the silhouette coefficient of the model; (4) select representative subcategories addressing different aspects of daily life for each ICF chapter (e1-e5); for each subcategory, identify specific terms, *t_i_ , t_j_,* extracted from their formal ICF definition, and run the word2vec model to generate the nearest semantic terms to *t_i_,* and *t_j_*; validate the obtained nearest semantic terms using relevant published literature; and (5) apply the results to a specific subcategory of a chapter involved in a relevant use case in the field of rehabilitation directly involved in daily living.

## Methods

### Data Collection

Reddit data were collected from pushshift.io [26] by the pushshift.io API (application programming interface). pushshift.io is a website that stores all publicly available Reddit submissions and comments, allowing researchers to collect and share Reddit datasets for research purposes, with extensive publications in related research (eg, Lama et al [27]). In this paper, we used the pushshiftr R package as a wrapper for the pushshift.io API [26].

### Word2vec

For training the word2vec model, we used the wordVectors R package [28]. It implements the original C code for word2vec [20].

### Semantic Distances

Given a vectorial representation of 2 words, their semantic similarity (S) was calculated using the cosine similarity measure between their respective vectorial representation, S(v1,v2). The semantic distances between 2 words, d(v1; v2), was calculated as 1 minus the semantic similarity, d(v1; v2)=1–S(v1; v2) [28].

### Semantic Categorization Test

In this test, we measured the capabilities of the model to represent the semantic categories based on the Battig and Montague category norms, an invaluable tool for researchers in many fields, with a recent literature search revealing their use in over 1600 publications in more than 200 different journals [29].

In this study, we use Van Overschelde’s [29] updated and expanded version of the Battig and Montague norms (expanded from 56 to 70 semantic categories).

In order to measure how well a word *i* is grouped in relation to the other words in its semantic category, we used the silhouette coefficients, *s(i).*

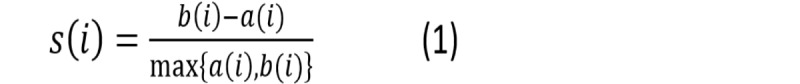



*a(i)* is the mean distance of word *i* with all other words within the same category, and *b(i)* is the minimum mean distance of word *i* to any words within another category (ie, the mean distance to the neighboring category). In other words, silhouette coefficients measure how close a word is to other words within the same category compared to words of the closest category [23].

## Results

### Sample Description

Data were collected from 20,344 submissions and 96,314 comments from the disability subreddit.

Total comments were posted by 10,411 Redditors, and the total submissions were posted by 9658 Redditors.

The total number of different Redditors that have posted a submission or a comment is 15,072.

Considering that Reddit moderators remove a percentage of submissions (eg, for not following the Reddit posting guidelines), the number of different Redditors (15,072) is quite close to the 17,545 subscribers presented in Figure 1.

The first comment on the disability subreddit included in this analysis was published in February 2009, and the last was published in December 2019.

Note that these data were publicly accessible on Reddit and that no personally identifiable information is included in this study. The dataset is publicly available per request.

This paper focuses on the analysis of the 96,314 comments; Multimedia Appendix 1 presents further details on the retrieved data. For example, the most common 10 words are *people* (appearing 4231 times), *disability* (3619), *time* (3418), *disabled* (2479), *feel* (2457), *lot* (2433), *person* (2264), *life* (2162), *day* (1841) and *job* (1811). Multimedia Appendix 1 also features a plot of the percentage of comments containing specific words (eg, *anger, hope, change, education*) by year, the fastest-growing words, and the words with the steepest increase in past years.

### word2vec

We then ran the *train_word2vec* function of the wordVectors R package to train the model, with the following parameters: vectors=200, threads=4, window=12, iter=5, negative_samples=0.

We performed a preliminary validation using a reduced subset of the original Mikolov paper analogies [20]. We selected only some of those that might fit in our context; therefore, we did not include, for example, the well-known analogy

king–man+woman=queen

but we obtained promising results in a variety of analogies, such as

brother–sister+husband=wife(0.352) (cosine distance is shown in brackets)

This means that if, for our trained model, we execute the *nearest_to* function as follows:

*nearest_to*(model[[“brother”]]-model[[“sister“]]+model[[“husband”]],5)

then we obtain “wife” as one of our top 5 nearest terms. We obtained several of them, with promising results. For example:

usa–ny+france=paris(0.666)

she–he+women=men(0.332)

doctor–hospital+teacher=school(0.599)

morning–woke+night=sleep(0.630)

girls–boys+women=men(0.474)

breakfast–8am+8pm=dinner(0.469)

cnn–news+netflix=streamer(0.515)

### Semantic Categorization Test

For the first 5 words of each of the first 65 semantic categories of the updated version of the Battig and Montague norm [29], we calculated the silhouette coefficients. The complete list of the 325 words and silhouette calculation is presented in Multimedia Appendix 2.

Multimedia Appendix 3, which shows the silhouette coefficients for the first 60 semantic categories, displays promising results. The numbers identifying each category are those presented in the original norm; for example, the first row is “3. A relative” because this is the third semantic category presented in the norm.

When analyzing the lower silhouette scores, we identified remarkable reasons for the miscategorization of the terms. For example, regarding the first semantic category, “1. A precious stone,” we selected *i*=diamond, and our model identified the category “43. A vegetable.” To be closer to *i* than category 1, we tried to find out why broccoli is closer to diamond than, for example, a ruby, and we found that diamond is a well-known class of broccoli. Similar explanations can be found for the other miscategorizations; for example, for category “59. A liquid,” we obtained *a(i)*=0.490 for *i*=water, but *b(i)* is lower [*b(i)*=0.381] because it is obtained for category “38. A non-alcoholic beverage.” This is because water is the first term in category 38 and is included in the mean distance calculation for *b(i),* (distance=0), but it is not included in the calculation of *a(i)* because the term *i* is not included in any calculation of *a(i)* (Multimedia Appendix 2). The same situation occurs with the category “20. An alcoholic beverage.”

It is important to note the lowest silhouette we obtained, *s(i)=-*0.645, for *i*=tuna in the “52. A fish” category (Multimedia Appendix 3). In this case, *b(i)* is remarkably lower because the distances to category “43. A vegetable” are lower for all words in the category. This means that our model finds a closer relation between, for example, broccoli or lettuce with tuna (a healthy diet) than between tuna and shark, bass, beta, or cod, which are very difficult to relate only to fish names.

In spite of obtaining some low silhouette codes, the detailed analysis led to promising results. Table 1 shows the algebraic operations (eg, food+cholesterol) that we used to focus the closest terms onto the specific ICF subcategories, avoiding the miscategorization problems.

**Table 1:** Products and technology (e1).

2^nd^ order code, 3^rd^ order code, and algebraic operations			Closest terms (cosine distance)
**e110 food and drugs**			
	**e1100 food**		
		food+cholesterol	fruits (0.347), veggie (0.372), wheat (0.382), broccoli (0.413), oatmeal (0.413)
		celiac+analysis	gluten (0.462), mitochondrial (0.510), coronary (0.513), psoriasis (0.519), genetic (0.526)
	**e1101 drugs**		
		drug+medicinal	marijuana(0.342), opioid(0.348), thc^a^(0.433), benzos(0.498), cbd^b^(0.451), wellbutrin(0.504)
**e115 assistive products**			
	**e1151 specially designed**		
		voice+controlled	siri(0.400), dragon(0.406), alexa(0.437)
		speech+app	voiceover(0.366), speechyfy (0.374), zoomtext(0.386)
**e120 mobility transportation**			
	**e1201 indoor outdoor**		
		motor+outdoor+power	powerchair (0.465), lightest (0.477), reclining(0.482), Invacare (0.502), wijits(0.506), pushrims(0.501)
		transfer+indoor+device	hoist(0.434), stow(0.439), hoyer (0.449), ultralight(0.497)
		taxi+wheelchair	uber (0.396), lyft (0.406), paratransit (0.420)
**e140 culture recreation and sport**			
	**e1401 adapted equipment**		
		adapted+sport	kayaking(0.389), archery (0.401), boccia (0.437),
**e150 routing**			
	**e1501 outdoor wayfinding**		
		wheelchair+apps	ableroad(0.541), wheelmap (0.551)

^a^thc: tetrahydrocannabinol.

^b^cbd: cannabidol.

### Mapping to the ICF’s Environmental Factors

Table 1 presents the closest terms to the representative subcategories of the products and technology chapter of the Environmental Factors. We present specific terms (eg, food and cholesterol, as illustrative examples) to show the potential of the model to discover relevant terms. In order to validate the results from Table 1, we went through the closest terms for each subcategory and identified recent publications and evidence.

The first terms for e1100, fruits and vegetables (veggie), have been extensively reported to be related to LDL (low-density lipoprotein) cholesterol [30], as well as wheat, oat [31], and broccoli [32].

We then considered other aspects related to food [eg, celiac disease (CD)]; we obtained *gluten* as the closest result, followed by *mitochondrial* (eg, reported by Picca et al [33]). The prevalence of the third term, c*oronary* *artery disease* (CAD), increases nearly twofold in patients with CD, as reported by Gajulapalli et al [34].

Ungprasert et al [35] demonstrated a significantly higher risk of CD among patients with psoriasis (fourth closest term) as well as genetic factors [36].

In relation to e1101 (drugs) cannabis, there is an increasing interest in the medical use of it (eg, in chronic pain, which is very common in disability) [37].

In relation to assistive products (eg, related to speech and voice), several state-of-the-art solutions were retrieved, such as Speechify, Zoomtext, Voiceover, Siri, Dragon, and Alexa.

In relation to e120, mobility transportation, desirable properties for outdoor transportation were retrieved, such as *lightest* or *reclining*, as well as top product providers (eg, Invacare and wijits). For indoor transportation, the closest terms were *hoist, stow,* and *hoyer*, as opposed to *powerchair,* which was retrieved for outdoors.

We then explored transportation services (taxi+wheelchair), and the closest terms were *uber, lyft,* and *paratransit*. It is important to remark that Uber [38] and Lyft provide specific disability policies [39].

In relation to e140, culture recreation and sport, several paralympic well-known ports emerged, such as *kayaking* or *archery*. Another close term was *boccia*, which is another (but perhaps less popular) Paralympic sport [40].

Finally, in relation to e1501, outdoor wayfinding, the wheelchair+app operation retrieved *ableroad* [41] and *wheelmap* [42] as closest terms.

Multimedia Appendix 2 includes a similar analysis for chapters e2-e5.

### Rehabilitation Use Case: Resilience (ICF e398 Subcategory)

As shown in Multimedia Appendix 2, for the e398 support and relationships subcategory, the closest term identified by our model is *resilience*. Resilience has been recently regarded as a key feature of rehabilitation and living life well following a disability [43]. Evidence shows that resilience-based skills have multiple benefits once applied to people’s lives (eg, a carry-over effect to other life domains). People do not have to be born resilient to become resilient; it can be improved with intentional practice [44].

It is unclear how well resilience or strategies to cultivate resilience are currently promoted as a component of rehabilitation programs. Furthermore, it does not yet have one unified definition, and research scholars have not decided on one specific understanding of what resilience means. Understanding resilience is an important component of building resilience [43].

Therefore, we used our model to identify its closest terms, and then we linked those terms to relevant publications. This is proposed as a straightforward example of how it can contribute to the understanding of a relevant term in rehabilitation. *Maturity, compassion, anger, resentment, grief, insecurity, contentment,* and *resenting* were identified by our model as the closest terms to *resilience*. Resilience and maturity have been extensively reported on (eg, by Davies et al [45]). The practice of compassion has been highlighted as an “essential component in nurturing resilience” [46]. As reported by Baldachino et al [47], the inability to cope effectively with anger may negatively impact a patient’s physical and psychological well-being in the realm of resilience. Designing evidence-based interventions aimed at decreasing the negative impact of anger on resilience can be advanced by examining the potential mediation effect between anger and resilience [48].

As noted by Howard and Meichenbaum [49], resentment is a way of undermining resilience; resentment is a form of chronic, deep-seated anger. Holding onto resentment and not letting it go can have deleterious health effects and undermine the development of resilience. In relation to grief, it has also been related to resilience (eg, Bonano et al [50]).

As was presented in the EU Social Insecurities and Resilience Report [51], people who have low levels of social insecurities more often report high levels of resilience. In recent psychological research [52], contentment was also directly associated with both resilience and life satisfaction and mediated the relationship between these 2 aspects of well-being. Details and further analysis is presented in Multimedia Appendix 1.

## Discussion

### Principal Findings

In this paper, we proposed social media as the link between word embeddings and the ICF’s environmental factors in a General Public License (GPL) framework (R-3.5.1). We applied a set of publicly available R libraries for collecting, model training, and analyzing Reddit public data. We trained a word2vec model using a small dataset and obtained encouraging results in king-queen–type analogies. Further, we obtained remarkable results in a standardized semantic categorization test. When mapping the discovered closest terms to representative subcategories of all ICF environmental factors, we verified them with scientific publications.

The obtained results open up interesting opportunities for exploration. Similarity isn’t just a way of finding the nearest words; it is also a way of extracting items of a single class in every environmental factor that makes up the physical, social, and attitudinal environment in which people live and conduct their lives, ranging from apps ”to know accessibility before you go“ to adapted sports (boccia). Therefore, it can be thought of as a form of topic modeling; however, rather than letting the algorithm choose a fixed number of topics, it gives us the option of choosing the specific term (such as *resilience*) and how expansive we want the explored space to be.

Medical professionals are currently being encouraged to participate in social media, as remarked upon by Stukus [53]; even if a health care professional is not interested in engaging in social media, they must still be aware of the information people may be encountering online in order to provide anticipatory guidance in the clinical setting [47]. Therefore, this study also intends to be a step in that direction.

### Limitations

The collected sample was not intended to be either representative or a comprehensive set of all comments posted by all persons with disabilities during the period under study. It includes all comments posted in Reddit’s disability subreddit; we did not include comments from other subreddits addressing specific disability causes (eg, stroke) because, in this study, we follow the approach of studying disability in general (instead of any specific group or etiology), an approach also adopted in other recent literature reviews (eg, Hästbacka et al [54]). In addition, the ICF is grounded in the principle of universality, namely, that functioning and disability are applicable to all people, irrespective of health condition. The ICF is committed to the principle of parity, which states that functional status is not determined by background etiology or, in particular, by whether one has a physical rather than mental health condition [9].

Furthermore, including only 93,614 comments allowed us to verify our hypotheses.

We did not include submissions in our analysis; we collected them (20,344 in total), but we did not use them for model training because only 2745 of them were labeled as “questions” while 1256 were labeled “articles and news,” 438 of them were labeled “videos,” and 13,570 were not labeled.

As presented in Multimedia Appendix 1, under the total number of comments by year, almost 80% of all comments took place during 2016-2019; therefore, the results of this study are weighted more strongly toward the recent years (2016-2019) rather than the early years (2009-2015) of the Disability subreddit.

Other relevant limitations to our study are related to the geographic location, spatial trajectory, or the time of day on which a comment was posted. As noted by Padilla et al [55] and Gore et al [56], such factors are relevant in social media. Spatiotemporal aspects are not controlled in our study; however, Reddit is most popular in the United States, with American users (representing 54% of Reddit’s user base) far outnumbering those from other countries. Second to the United States, the UK represents the next highest share of Reddit traffic, at 8%, with Canada rounding out third, at 6.4%. Reddit is most popular amongst young adults between the ages of 25-34 years, which make up more than half of the site’s users. Nevertheless, Reddit still draws in a large number of middle-aged users. A 2016 study found that people between the ages of 30-49 years accounted for 33% of the site’s users, indicating that Reddit is a viable platform for reaching both young and middle-aged adults. Reddit is particularly popular among males, who make up more than two-thirds of the site’s users [16].

### Comparison with Prior Work

In medical applications, word2vec has recently been applied to larger datasets, such as 641,279 French health-related documents produced in a professional context (Dynomant et al [57]), 880,165 papers in a biomedical publication venue (Feng et al [58]), 1,451,413 abstracts for Adverse Drug Event Discovery Using Biomedical Literature (Tafti et al [59]), and 1,749,870 reviews in Online Doctor Reviews [60].

Reddit has recently been used as a data source for studies in chronic diseases, such as Foufi et al’s [13] analysis of 17,624 text posts for entity and relation extraction, employing the PKDE4J tool. Sharma et al [61] performed a qualitative analysis of Reddit discussions regarding motivations and limitations associated with vaping among people with mental illness, a thematic analysis that included 3263 comments from 133 discussion threads.

In a small corpus (37,000 and 140,000 documents) using a semantic categorization test, word2vec was applied in analyzing and disambiguating the content of dreams [62]; this research field addresses questions such as “how do gender, cultural background, and waking-life experiences shape dream content?”.

Facebook groups, discussion forums, and chat rooms were recently analyzed [63] to explore and compare the interactions and connections among online support groups to better understand how people with disabilities were utilizing different social networks to facilitate communication interchange. They concluded that through participation on different platforms, persons with disabilities are able to provide and receive social support in various ways, without the barriers and constraints often experienced by this population.

Our approach is completely different in that we provide a tool for discovering terms of interest (in this paper, we applied it to the ICF environmental factors, but it could also be applied to, for example, the ICF’s body functions and structures). For example, regarding e5 services and associations, in using the 3 terms *hear+association+kid,* we obtained *depaul* as one of the closest results. The DePaul School for Hearing and Speech teaches children who are deaf or hard of hearing to listen and speak without sign language [64]. Another obtained close term is *saskatoon*, a new early learning pilot program in Saskatoon that impacts preschoolers who are deaf or hard of hearing by breaking down communication barriers [65].

Therefore, it can be addressed to a wide variety of involved stakeholders besides people with disabilities themselves, such as informal caregivers, health care professionals, and private or public associations.

### Conclusions

This study explored the ability of word2vec to extract the main factors affecting the lives of people with disabilities within the ICF framework from a small dataset, showing promising results.

Our results open up interesting opportunities for exploration and discovery. Similarity is revealed as not only a way of finding the nearest words but also a way of extracting out items related to specific elements. Therefore, it can be thought of as a form of topic modeling, where users can focus on a particular term in-breath or in-depth.

## Appendix

Multimedia Appendix 1 Word trends, closest terms, environmental factors, and resilience.

Multimedia Appendix 2 Semantic Categorization Test.

Multimedia Appendix 3 The 60 semantic categories ordered by silhouette value.

## Abbreviations

ADLs: aspects of daily living
API: application program interface
BERT: bidirectional encoder representations from transformers
CBD: cannabidiol
CBOW: continuous bag of words
EF: environmental factors
ICF: International Classification of Functioning Disability and Health
LDL: low-density lipoprotein
NLP: natural language processing
THC: tetrahydrocannabinol
WPIE: whole post integrity embeddings
