# Supplementary material for: Discovering the Context of People With Disabilities: Semantic Categorization Test and Environmental Factors Mapping of Word Embeddings from Reddit
Source: JMIR Med Inform. 2020 Nov 20;8(11):e17903. doi: 10.2196/17903 (PMC7718084; doi:10.2196/17903)
Supplement: Multimedia Appendix 1 [file medinform_v8i11e17903_app1.docx]

**Disability Subreddit statistics**: Number of suscribers by year


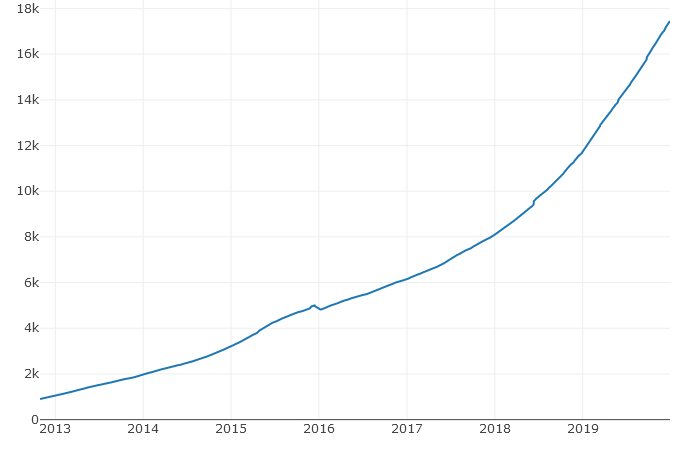


Comments per day


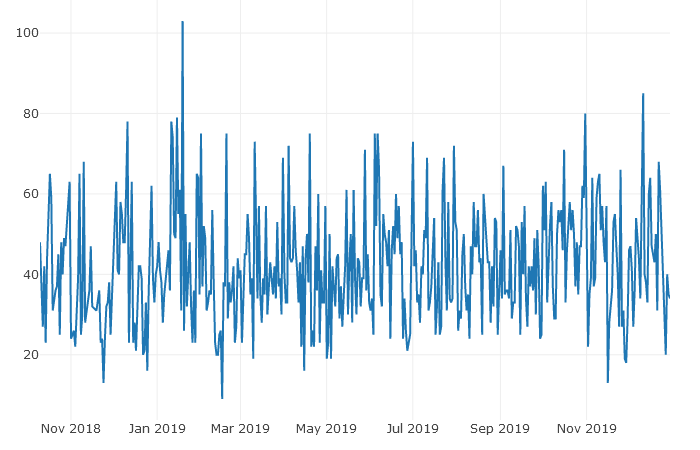


Extracted from

<https://subredditstats.com/r/disability>

The following plots are not extracted from <https://subredditstats.com/r/disability>

They have been generated by the authors,


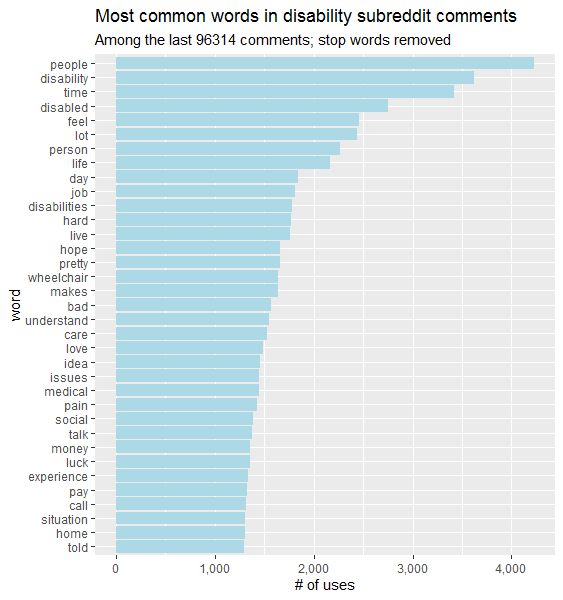


**Percentage of comments containing specific words by year**


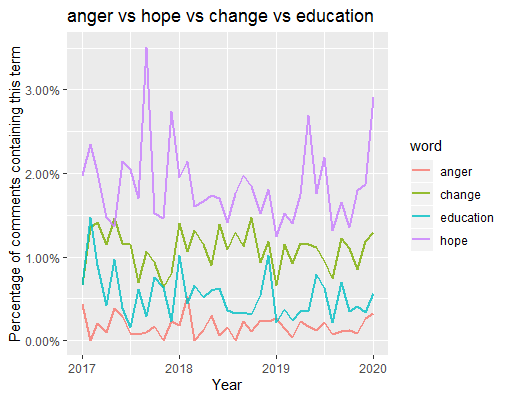


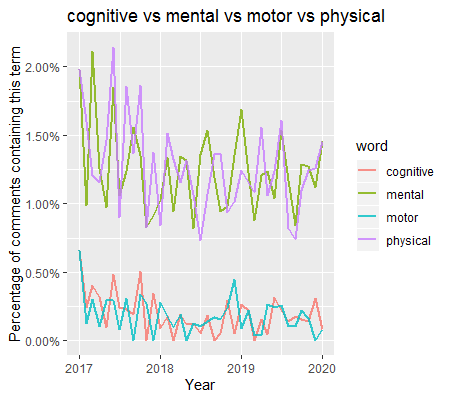


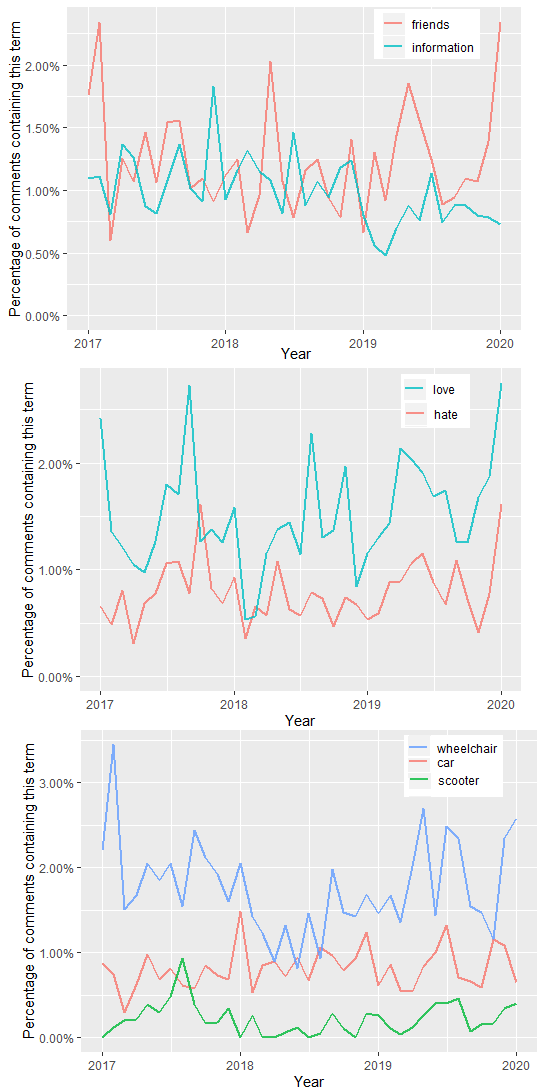


**Fastest growing words**


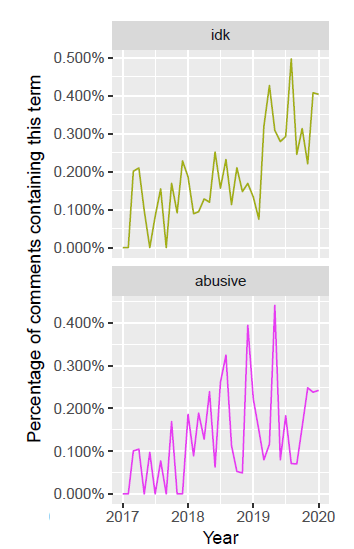


word term estimate std.error statistic p.value

1 idk year 0.528 0.117 4.50 6.85e- 6

2 plastic year 0.368 0.123 2.98 2.91e- 3

3 beautiful year 0.362 0.104 3.48 4.99e- 4

4 abusive year 0.345 0.134 2.58 9.79e- 3

**IDK Support 4 You** is a service for people (including older people, people with learning disabilities, physical impairments and/or sensory impairments, people with mental health issues, children and people in transition).

**Closest terms to e2..e5 Environmental Factors**

**Table SM1**. Natural environment (e2) Support and relationships (e3), attitudes (e4) and services, systems and policies (e5)

| Code | ICF code | operation | Closest terms (distance) |
| --- | --- | --- | --- |
| e220 flora and fauna | e2201 animals | animals | dogs(0.236), pets (0.345), esa (0.336), cats (0.421), horses (0.464), rabbits (0.425), sd (0.510) |
| e250 sound | e2500  sound intensity | noise | cancelling (0.278), headphones (0.306), flashing (0.475), vibrating (0.502) |
| e390 emotional support | e399 excluding domestic animals | emotional + support - animals | resilience (0.628), compassion (0.630), resentment (0.635), stigma (0.644), openness (0.655), challenges (0.659) |
| e420 individual attitudes | e420 negative from friends | stigma + friends | hatred (0.572), preconceptions (0.576), jokes (0.577), isolation (0.583), loneliness (0.584), cripples (0.593) |
|  | e430 negative from superior | stigma + boss | hostility (0.535), discrimination (0.548), ignorance (0.557), ableism (0.598), mindset (0.599), detriment (0.603) |
|  | e465 negative from ideologies | stigma + ideology | pervasive (0.385), innately (0.412)  prejudice (0.432), rationalization (0.442), deshumanization(0.443) |
| e525 housing | e5250 locating, providing and maintaining | housing + benefitis | hud (0.279), subsidized (0.288), vouchers (0.373), liheap (394), hcv (0.397), pha (0.435), stamps (0.455) |
| e550 legal services | e5502 legal policies | benefits + services | dfs (0.396), hhs (0.410), dpss (0.454), fafsa (0.468) |

esa: emotional support animal, sd: service dog, hud: Housing and Urban Development, liheap: Low Income Home Energy Assistance Program, hcv: Housing Choice Voucher, pha: Philadelphia Housing Authority, dfs: Department of Financial Services, hhs: Department of Health and Human Services, dpss: Department of Public Social Services, fasfa: Free Application for Federal Student Aid

In Table SM1, the closest terms to e2201 animals, are *dogs*, followed by *pets* and *esa* (emotional support animal). An *esa* differs from a service animal. Service animals are trained to perform specific tasks, such as helping a blind person navigate as in the case of service dog (*sd*, also identified as one of the closest terms in Table SM1 for this category ), while *esas* receive no specific training.

When we analyzed e390 emotional support, we had to exclude *animals* (emotional + support - animals ) because when searching only emotional + support, several closest terms were related to animals, specially dogs. The first term here is resilience which we will analyze specifically in the next section, as well as *compassion* and *resentment* which are indeed related to emotional support*.*

In relation to e420 individual attitudes, we present 3 representative subcategories (e420 negative from friends, e430 negative from superior and e465 negative from ideologies) because the closest terms are remarkably different in the 3 cases.

When negative attitudes come from friends, *isolation* and *loneliness* are among the closest. This aspects have been specifically reported in a recent study conducted in UK by MacDonald et al, entitled *‘The invisible enemy’: disability, loneliness and isolation*, where they concluded that “the experience of social isolation and emotional loneliness increased significantly when analyzing data on disability.

When negative attitudes come from superior, *ableism* is among the closest terms. As recently reported by Jammaers et al, ableism has recently been advanced as a new lens to conceptualize the marginalization of disabled people at work.(Jammaers 2016).

When negative attitudes come from ideology, *prejudice* and *deshumanization* are among the closest terms. Findings indicate that strong negative emotional reactions to outgroups (i.e., revulsion, disgust) are associated with dehumanization; moreover, mediational analyses suggested that dehumanization fed into prejudice attitudes, thus, prejudice toward people with developmental disabilities would be rooted in dehumanization (Parker 2018).

When analyzing e5250 related to housing several acronyms, representing governmental organizations and services are reported in Table SM1. Some of them are *hud* (Housing and Urban Development), *liheap* (Low Income Home Energy Assistance Program), *hcv* (Housing Choice Voucher), *pha* (Philadelphia Housing Authority).

Similarly, for e5502 legal policies, dfs (Department of Financial Services), hhs ( Department of Health and Human Services), dpss (Department of Public Social Services), fasfa (Free Application for Federal Student Aid).

Also we identified several numeric codes, not included in Table SM1, such as *620* which refers to the H.R. (House of Representatives) 620 ADA (American with Disabilities Act) Education and Reform Act of 2017. This reform has given rise to controversy during 2018, as it would *update the law for disabled Americans for modern times, say Republicans, meanwhile Democrats say it would set disabled Americans’ civil rights back decades.*

Other numeric codes that we have obtained and not included in Table SM1 are 733 which refers to-termination of temporary total disability social benefits, or 791 which refers to U.S.C. 791 United States Code Section 791 - Employment of individuals with disabilities.

**Resilience**

Resilience, as a generic concept is concerned with how individuals cope with stress and how they recover from trauma, promoting positive development, inclining towards the future and hope. Resilience was significantly associated with less hopelessness, (Somasundaram 2016) therefore we applied our model to *resilience* and *hopeless*.

We obtained the top 10 closest words to *resilience* and the top 10 closest words to *hopeless* with our model and created a hierarchy of them based on their similarities applying hierarchical clustering.

We applied hclust function (of the stats R package) with the complete linkage partition method to the matrix of dissimilarities obtained with the cosineDist function.

Therefore we obtained the cluster dendogram presented in Figure 2. The initial partition involves two main clusters, one represented by *resilience* and its related concepts and the other by *hopeless*, as expected, providing a straightforward initial validation of the method. For 2 clusters, the obtained Dunn index is 0.5528, Dunn index values larger than 0.50 are regarded as reasonable structures

As shown in Figure 2, maturity, compassion, anger, resentment, grieve, insecurity, contentment and resenting, have been identified by the model as closest terms to *resilience*.

Therefore in the following paragraphs till the end of this section we provide evidence relating such terms to *resilience*, in order to support our hypothesis that the model can in fact be used to increase our understanding of a term, leading us to related and relevant aspects of it. In this sense the model supports the generation of more specific topic models.


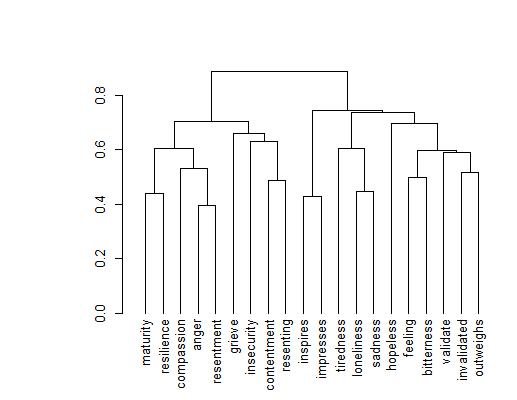


Figure 2. Cluster dendogram of the 20 closest words to resilience and hopeless

Resilience and *maturity* have been extensively reported, for example as remarked in the book Disability and Impairment: Working with Children and Families (Davies 2008), resilience helps to explain why when faced with apparently similar situations, people may react differently, this the situation of both siblings and children with disabilities when parents express the view ‘They grow beyond their years’, to explain the maturity and understanding that siblings of children with disabilities commonly share.

The practice of *compassion* has been highlighted as an “essential component in nurturing resilience” (Marini 2017), barriers to developing compassion are anger, fears and pitting others. They remark that compassion development involves acknowledging the fact that all individuals at some point will fell sorrow, disappointment and pain.

As reported by Baldachino and coallegues, the inability to cope effectively with *anger* may negatively impact a patient"s physical and psychological well-being in the realm of resilience (Baldacchino et al 2012). Designing evidence-based interventions aimed at decreasing the negative impact of *anger* on resilience can be advanced by examining the potential mediation effect between anger and resilience (Wu et al 2017). Self-concept has been identified as possible mediator, therefore the interaction between self-concept(mediator), *anger* (the independent variable), and resilience (the dependent variable) has the potential to expand knowledge and identify useful avenues for psychosocial interventions that could enhance the well-being (Wu 2017).

*Resentment* and *anger*, identified by disubreddit2vec as nearest words in the leftmost subtree in Figure 2, are in fact closely related. As remarked by Howard and Meichenbaum (2015), *resentmen*t is a way to undermine resilience. Resentment is a form of chronic deep-seated anger. Holding onto *resentment*, not letting it go can have deleterious health effects and undermine the development of resilience.

In relation to *grieve*, traditionally “absent grief” stems from denial or inhibition, and that it is generally maladaptive in the long run. Increasingly, however, investigators have challenged this assumption, arguing that some people do not show overt signs of distress because of quick adjustment following expected loss or because of personality factors that promote an inherent resilience to loss (Bonano et al 2002).

As presented in the EU Social Insecurities and Resilience Report (EUROFOUND) *insecurities* affect quality of life to a lesser degree if there are mechanisms that can cushion the negative impacts they might have. People who have low levels of social *insecurities* more often report high levels of resilience (Eurofound 2018).

*Contentment* , was directly associated in recent psychological research (Gerson, 2018). with both resilience and life satisfaction and mediated the relationship between these two aspects of well-being.

Finally *resenting* in this context can be interpreted as feeling *angry* because you have been forced to accept someone or something that you do not like.

Of course these publications are not uploaded directly by redditors in the disability subreddit, but it seems as if redditors comments somehow integrate (parts of) this knowledge, especially when it is directly related to their personal experiences
