# Supplementary material for: Discovering the Context of People With Disabilities: Semantic Categorization Test and Environmental Factors Mapping of Word Embeddings from Reddit
Source: JMIR Med Inform. 2020 Nov 20;8(11):e17903. doi: 10.2196/17903 (PMC7718084; doi:10.2196/17903)
Supplement: Multimedia Appendix 3 [file medinform_v8i11e17903_app3.docx]

| **Category** | **s** |
| --- | --- |
| 3. A relative | 0,562 |
| 29. A sport | 0.475 |
| 43. A vegetable | 0.429 |
| 10. A color | 0.402 |
| 55. A state | 0.323 |
| 49. A disease | 0.301 |
| 13. A part of speech | 0.285 |
| 40. A science | 0.254 |
| 27. An occupation or profession | 0.239 |
| 31. An article of clothing | 0.217 |
| 58. A type of car | 0.196 |
| 56. A college or university | 0.195 |
| 9. A type of fabric | 0.186 |
| 44. A type of footwear | 0.162 |
| 54. A city | 0.131 |
| 39. A transportation vehicle | 0.119 |
| 35. A kind of money | 0.116 |
| 4. A unit of distance | 0.113 |
| 57. A drug | 0.11 |
| 28. A natural earth formation | 0.104 |
| 46. A female first name | 0.098 |
| 8. A four-footed animal | 0.085 |
| 2. A unit time | 0.079 |
| 24. A member of the clergy | 0.077 |
| 19. A type of human dwelling | 0.075 |
| 18. An elective office | 0.074 |
| 32. A part of a building | 0.074 |
| 11. A kitchen utensil | 0.064 |
| 6. A type of reading material | 0.063 |
| 7. A military title | 0.063 |
| 25. A substance for flavoring food | 0.04 |
| 60. A thing women wear | 0.023 |
| 21. A country | 0.008 |
| 12. A building for religious services | -0.008 |
| 33. A chemical element | -0.028 |
| 15. A part of the human body | -0.029 |
| 1. A precious stone | -0.037 |
| 26. A fuel | -0.04 |
| 23. A carpenter's tool | -0.051 |
| 16. A fruit | -0.053 |
| 17. A weapon | -0.058 |
| 34. A musical instrument | -0.064 |
| 30. A weather phenomenon | -0.066 |
| 47. A male first name | -0.081 |
| 41. A toy | -0.092 |
| 51. A type of ship/boat | -0.092 |
| 37. A bird | -0.102 |
| 48. A flower | -0.118 |
| 36. A type of music | -0.132 |
| 22. A crime | -0.134 |
| 5.A metal | -0.137 |
| 14. An article of furniture | -0.16 |
| 38. A non-alcoholic beverage | -0.176 |
| 20. An alcoholic beverage | -0.212 |
| 59. A liquid | -0.223 |
| 50. A tree | -0.281 |
| 53. A snake | -0.316 |
| 45. An insect | -0.347 |
| 42. A type of dance | -0.365 |
| 52. A fish | -0.645 |
